# Supplementary material for: Activated Protein C Ameliorates Tubular Mitochondrial Reactive Oxygen Species and Inflammation in Diabetic Kidney Disease
Source: Nutrients. 2022 Jul 29;14(15):3138. doi: 10.3390/nu14153138 (PMC9370435; doi:10.3390/nu14153138)
Supplement: Supplementary file 1 [file nutrients-14-03138-s001.zip › supplementary-figures.pdf]

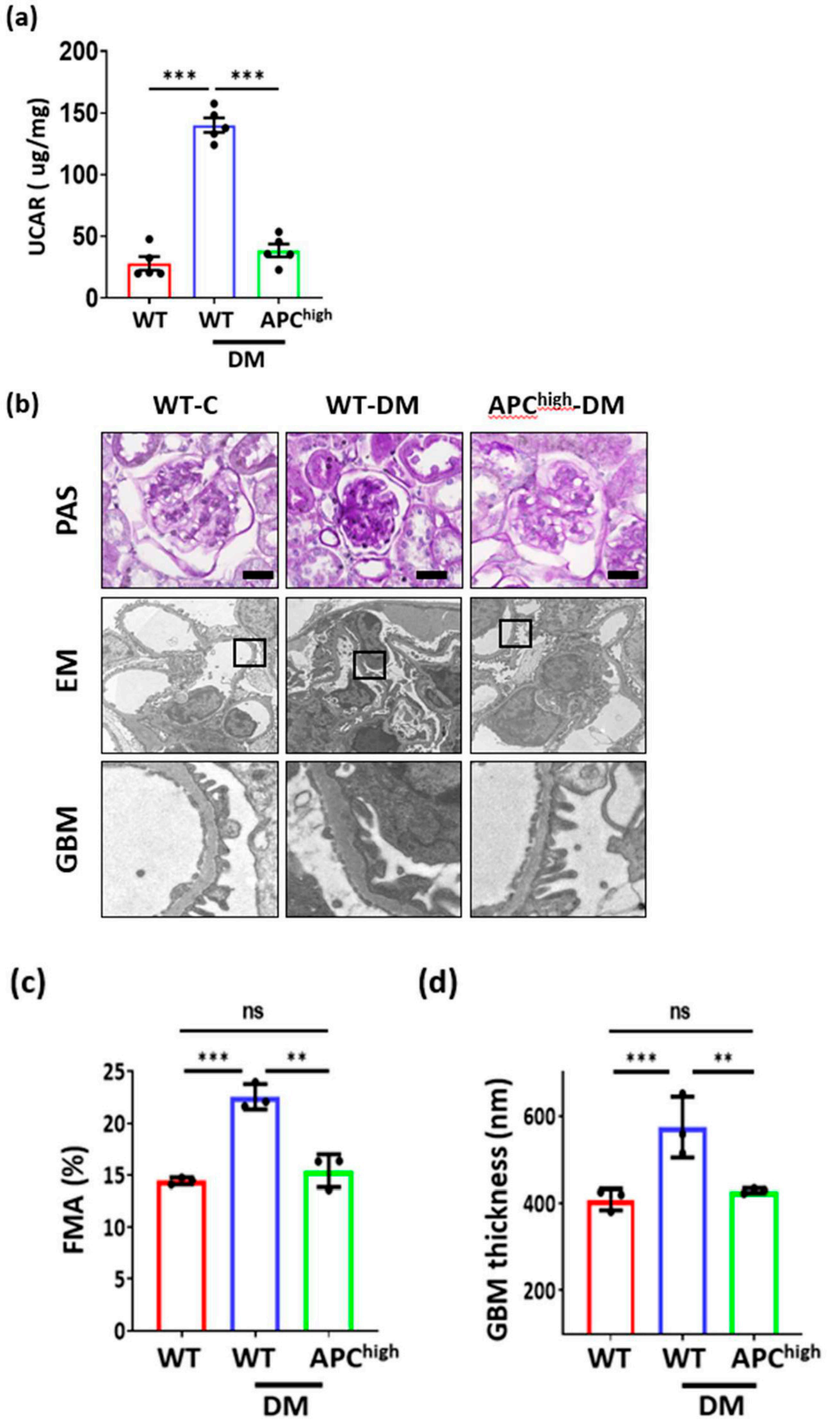

Supplementary Figure S1. (a) Albuminuria (UACR, urine albumin creatinine ratio) control nondiabetic (WT) and diabetic wild-type (WT-DM) or APC^{high}-DM mice after 24 weeks of hyperglycaemia; each dot represents data from one mouse. (b-d) Exemplary images of PAS-stained kidney sections (top row) and of the glomerular filtration barrier (middle and bottom row; transmission electron microscopy) and bar graphs with dot plots summarizing fractional mesangial area (e) and GBM thickness (glomerular basement membrane thickness, nm, d); groups as in a. Means ±SEMs of at least three mice per group. *P < 0.05; **P < 0.01; ***P < 0.001 (a, c-d). Statistical analyses were performed using one-way ANOVA with Sidak's multiple comparison test.

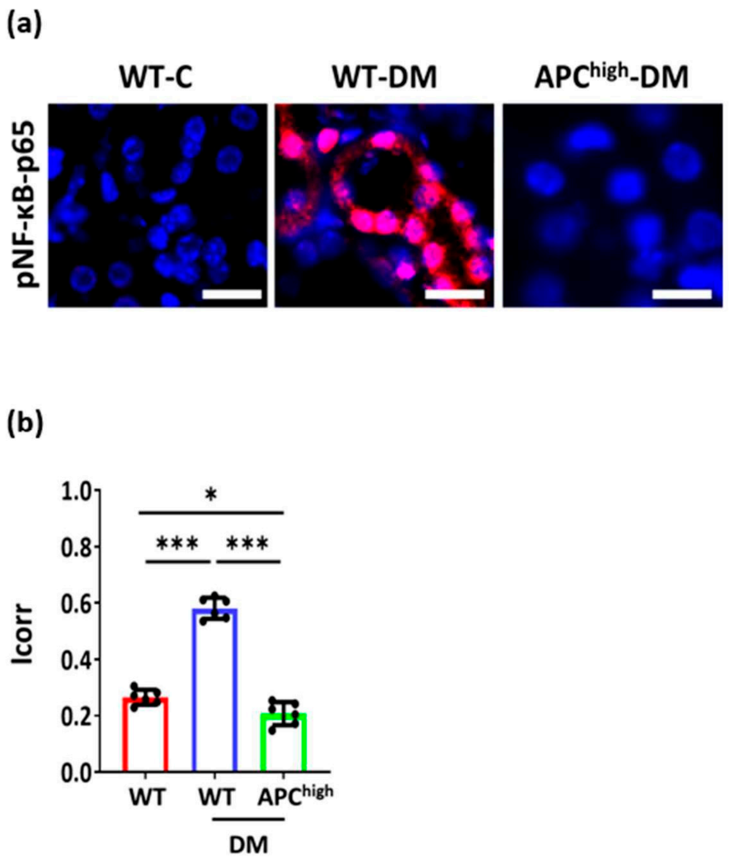

Supplementary Figure S2. Representative images (a) of immunohistochemical staining of p65-NF-κB (red; DAPI nuclear counter stain, red) and bar graph with dot plot showing Icorr-scores for p65-NF-κB and DAPI colocalization in mouse kidneys obtained from nondiabetic wild-type (WT-C), diabetic wild-type (WT-DM), or diabetic APC^{high} mice (APC^{high}-DM); scale bar: 20 µm; *P < 0.05; **P < 0.01; ***P < 0.001 (b). Statistical analyses were performed using one-way ANOVA with Sidak's multiple comparison test.
